# An artificial intelligence method to assess the tumor microenvironment with treatment outcomes for gastric cancer patients after gastrectomy

**DOI:** 10.1186/s12967-022-03298-7

**Published:** 2022-02-21

**Authors:** Tao Chen, Xunjun Li, Qingyi Mao, Yiyun Wang, Hanyi Li, Chen Wang, Yuyang Shen, Erjia Guo, Qinglie He, Jie Tian, Mansheng Zhu, Jing Wu, Weiqi Liang, Hao Liu, Jiang Yu, Guoxin Li

**Affiliations:** 1 Department of General Surgery & Guangdong Provincial Key Laboratory of Precision Medicine for Gastrointestinal Tumor, Nanfang Hospital, The First School of Clinical Medicine, Southern Medical University, Guangdong Provincial Engineering Technology Research Center of Minimally Invasive Surgery, No. 1838, North Guangzhou Avenue, Guangzhou, 510515 Guangdong China; 2 School of Biomedical Engineering, Southern Medical University, Guangzhou, 510515 Guangdong China; 3 Medical Image Center, Nanfang Hospital, Southern Medical University, Guangzhou, 510515 Guangdong China

**Keywords:** Gastric cancer (GC), Tumor microenvironment (TME), ImmunoScore (IS), Radiomic ImmunoScore (RIS), Artificial intelligence (AI)

## Abstract

**Background:**

The tumor microenvironment (TME) plays an important role in the occurrence and development of gastric cancer (GC) and is widely used to assess the treatment outcomes of GC patients. Immunohistochemistry (IHC) and gene sequencing are the main analysis methods for the TME but are limited due to the subjectivity of observers, the high cost of equipment and the need for professional analysts.

**Methods:**

The ImmunoScore (IS) was developed in the TCGA cohort and validated in GEO cohorts. The Radiomic ImmunoScore (RIS) was developed in the TCGA cohort and validated in the Nanfang cohort. A nomogram was developed and validated in the Nanfang cohort based on RIS and clinical features.

**Results:**

For IS, the area under the curves (AUCs) were 0.798 for 2-year overall survival (OS) and 0.873 for 4-year overall survival. For RIS, in the TCGA cohort, the AUCs distinguishing High-IS or Low-IS and predicting prognosis were 0.85 and 0.81, respectively; in the Nanfang cohort, the AUC predicting prognosis was 0.72. The nomogram performed better than the TNM staging system according to the ROC curve (all P < 0.01). Patients with TNM stage II and III in the High-nomogram group were more likely to benefit from adjuvant chemotherapy than Low-nomogram group patients.

**Conclusions:**

The RIS and the nomogram can be used to assess the TME, prognosis and adjuvant chemotherapy benefit of GC patients after radical gastrectomy and are valuable additions to the current TNM staging system. High-nomogram GC patients may benefit more from adjuvant chemotherapy than Low-nomogram GC patients.

**Supplementary Information:**

The online version contains supplementary material available at 10.1186/s12967-022-03298-7.

## Introduction

The tumor-node-metastasis (TNM) staging system is widely used in the classification of cancer; is based on the primary tumor (T), regional nodes (N) and metastasis (M); and helps in choosing surgery, chemotherapy, etc. However, recent studies have shown that the TNM staging system does not perfectly predict the prognosis of gastric cancer (GC) patients [1]. Those who had the same TNM stage and received similar treatment had widely varied clinical outcomes [2, 3]. As increasing evidence has indicated the critical forecasting capability of immune infiltration in GC patients [3], having immune cell markers along with TNM staging may help.

There is reportedly an exact correlation between tumor and immunocyte infiltration of the tumor microenvironment (TME) [4], which might lead to different prognoses of GC patients. In addition, the evaluation of the immune microenvironment seems to be useful in predicting survival and chemotherapy response for patients [5]. Therefore, specific analysis of an individual's tumor microenvironment and targeted selection of therapy regimens based on the assessment will be considerably helpful for patients.

Because the importance of immunocyte infiltration of the TME has been recognized, ways in which to analyze the immune microenvironment have been developed. To analyze the types and composition of immunocytes in the TME, biopsies were initially analyzed using immunohistochemistry (IHC) [3], which is quite accurate and realistic [6]. However, to a certain extent, the interpretation of IHC results depends on the subjectivity of the observer. Another method of determining immunocyte infiltration of the TME uses indirect information about the composition of immunocytes that can be calculated by gene expression profiles [7], which was proven to be effective. Because the calculation process of the results is traceable and easy to repeat, the results might be more objective.

Although calculation by gene sequencing is already objective, it still has some limitations, including a high cost for patients and specialized gene sequencing equipment for hospitals. Medical imaging may be a viable solution to this problem. Medical images are more than pictures, but data [8], such as radiomics, have been developed and successfully applied in many fields. Furthermore, many scholars have found that some tumor imaging features are closely related to tumor gene expression, providing a new idea for survival prediction and clinical strategy adjustment by combining their analyses [9, 10]. For evaluating the tumor microenvironment of GC patients, radiomics enables the extraction of innumerable quantitative features from medical images with high-throughput computing [11] and the establishment of a model. Several studies have explored the association between imaging features and tumor-infiltrating lymphocytes, such as CD8 + cells [12].

Supervised machine learning (ML) is a type of artificial intelligence that is widely used and shows great potential in precision oncology [13, 14]. As a mature machine learning method, support vector machines (SVMs) have great potential in solving cancer prediction problems based on diverse and complex clinical data [1, 15].

In this study, we analyzed the relationship between the gene expression the and CT images of GC patients to assess immunocyte infiltration of the TME based on the SVM model and developed an excellent nomogram based on clinicopathological factors and the SVM model to predict survival and chemotherapy benefit in GC patients.

## Methods and materials

### Study design and patients

**Fig. 1:**
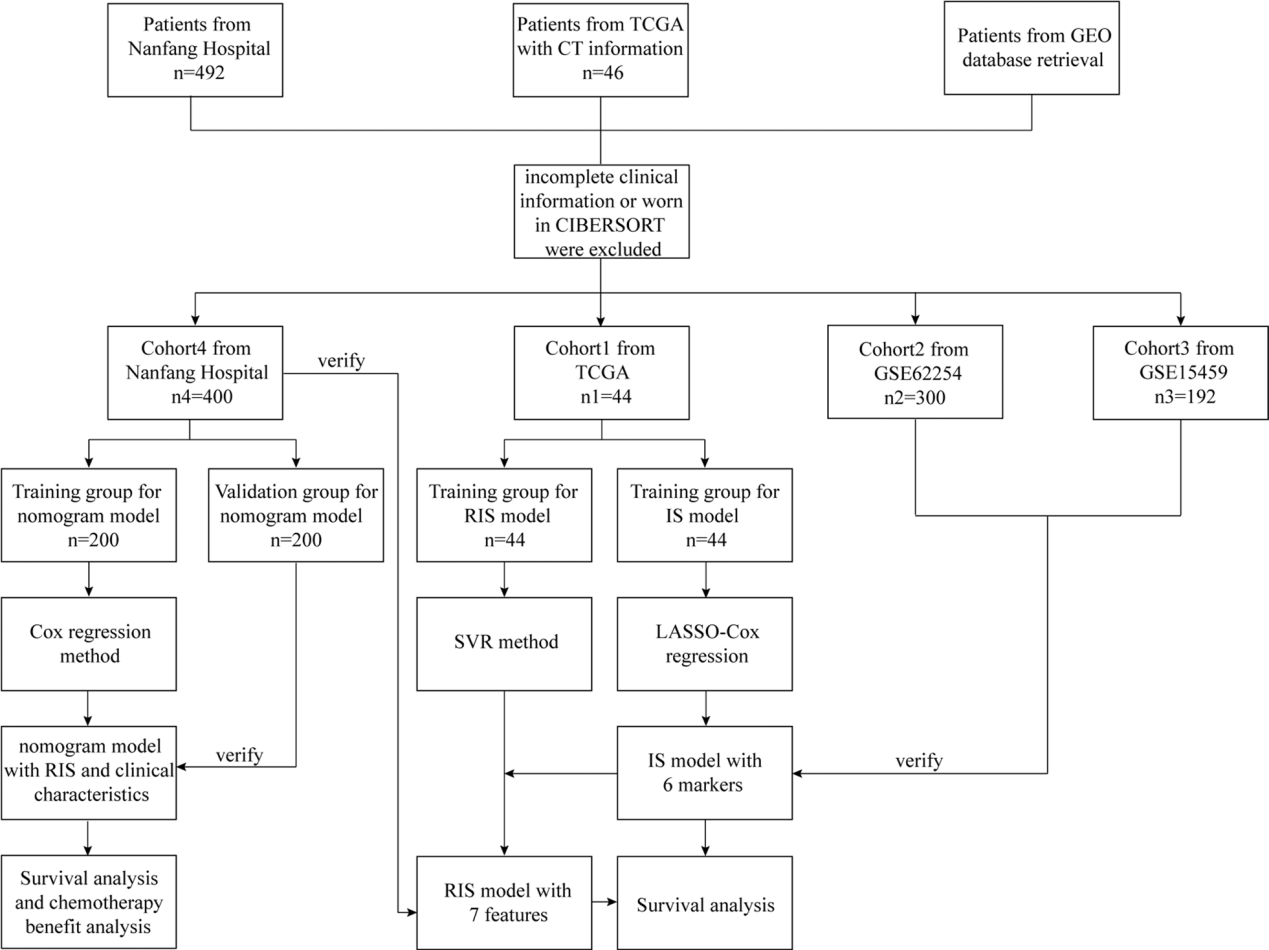
Flow chart of the whole study design, including data collection and analysis

The design of the entire study is shown in Fig. 1. A total of 4 independent cohorts, which included 936 GC samples, were enrolled in this study. One cohort (n 1 = 44) was from The Cancer Genome Atlas (TCGA) datasets (https://portal.gdc.cancer.gov/) and 2 cohorts (GSE62254, n2 = 300, and GSE15459, n3 = 192) were from Gene Expression Omnibus (GEO) datasets (https://www.ncbi.nlm.nih.gov/geo/), and a cohort (n4 = 400) from Nanfang Hospital (Guangzhou, China), which were used to analyze the relationship between gene expression and immune microenvironment of tumors. We developed an SVM model to analyze the relationship between gene expression and CT images based on a TCGA cohort, aiming to learn the immune microenvironment from CT images. CT images were obtained from The Cancer Imaging Archive (TCIA, https://www.cancerimagingarchive.net/). One cohort that comprised 400 consecutive patients from Nanfang Hospital (Guangzhou, China) from October 2004 to September 2011 was used to develop the nomogram to predict survival and chemotherapy benefit for GC patients. All of these patients underwent partial or total radical gastrectomy. The inclusion criteria were histologically confirmed GC and patients who underwent standard unenhanced and contrast-enhanced abdominal CT < 30 days before surgical resection. Those who received preoperative chemotherapy, had severe surgical complications, did not have complete follow-up data over 3 years, or had other coexisting cancers were excluded. The entire study design is shown in Fig. 1.

The TNM staging was reclassified, and the postoperative chemotherapy regimen was taken based on the guidelines of the National Comprehensive Cancer Network (NCCN) [16]. In the Nanfang Hospital cohort, up to 223 patients were treated with adjuvant chemotherapy, including 114 (51.1%) patients treated with the XELOX (capecitabine-oxaliplatin, capecitabine 1000 mg/m^2^/days day1–day14, oxaliplatin 130 mg/m^2^ once) regimen, 90 (40.4%) patients treated with the FOLFOX (fluorouracil-folinic acid-oxaliplatin, fluorouracil 400 mg/m^2^ at day1 and 2400–3600 mg/m^2^ for 46 h, folinic acid 400 mg/m^2^ once, oxaliplatin 85 mg/m^2^ once) regimen and 19 (8.5%) patients treated with 5-FU treatment alone or other regimens.

The follow-up duration was calculated from the day of surgery to the last follow-up. We defined the time to all-cause death as overall survival (OS) and defined the time to tumor recurrence at any site or death as disease-free survival (DFS). Ethical approval was obtained for this retrospective study, and informed consent was obtained from the patients.

### Assessment of immunocyte infiltration of the TME and calculation of the ImmunoScore (IS)

To predict the prognosis of patients using gene information to assess immune infiltration, we included 44 cases from TCGA as the training cohort, 300 cases from GSE62254 and 192 cases from GSE15459 as 2 validation groups to establish and evaluate the role of the ImmunoScore (IS) in clinical prognosis.

To quantify the proportions of 22 types of infiltrating immunocytes of the TME, normalized RNA sequencing data were analyzed using the CIBERSORT algorithm [17]. The immunocyte type fractions of patients in the training cohort using the CIBERSORT algorithm could be downloaded directly from a comprehensive resource [18] (http://timer.cistrome.org/). For each sample, the sum of all estimates of the immunocyte type component equaled 100%. The least absolute shrinkage and selection operator (LASSO) method was used to screen the most effective prognostic markers among 22 immune cell subsets, and the optimal values of the penalty parameter λ were determined by 20-fold cross-validations.

The ImmunoScore (IS) was calculated by a Cox proportional hazards model with the LASSO results. The output was expressed as coefficients for estimating the immunocytes of the TME. The calculation string formula was as follows:

$${\text{IS}} = \alpha 0 + \sum {\beta_{\text{i}} }{\text{X}}_{\text{i}},$$

where X_i_ represents the relative component of each immunocyte, β_i_ is the regression coefficient for the predictor and α is often a constant. We divided GC patients into 2 groups (High-IS and Low-IS) based on the median IS in each cohort.

### Exploration between ImmunoScore (IS) and immunocytes and immune checkpoint

Differential expression gene (DEG) analysis of the GSE15459 cohort was performed by applying the “limma” package at a corrected P < 0.05 and | logFC |≥ 1.5. The resulting data were used to generate volcano plots using R software (version 4.0.4). Data on the abundance of 22 types of immune infiltrates from the TIMER website were obtained. Using the “ggplot 2” package, we identified immunocytes with significant differences between the High-IS and Low-IS groups while analyzing the correlation between these cells and the IS using the “ggstatsplot” package.

### CT image processing, feature extraction and development of the radiomic ImmunoScore (RIS)

To use CT information to obtain radiomics features associated with the IS of patients, we included 44 cases from TCGA as the training cohort and 400 cases from Nanfang Hospital as the validation cohort to establish a Radiomic ImmunoScore (RIS) to predict IS and estimate its connection with patient survival.

Using ITK-SNAP software (www.itksnap.org, version 3.8), two radiologists manually delineated the primary tumor on the CT images. The CT number, representing the absorptivity of tissue to X-rays, was set ranging from − 150 to − 50 Hounsfield units to exclude bone, muscle, blood vessels, and other intra-abdominal organs [19, 20]. The region of interest (ROI) was delineated by hand along the gastric wall at a distance of 1 mm from the stomach wall around the tumor lesion.

Based on extracted image features and ImmunoScore, the RIS model was developed by SVM, aiming to predict the ImmunoScore by CT images. The common image features included color, texture, shape and spatial relation. The software version we used was MATLAB 2018a, and the shape of the feature matrix extracted from a CT image was 1*9694. In the training cohort, the size of the original CT feature matrix was 44*9694, which made regression difficult. Therefore, the average weight of each feature was finally obtained after dimensional reduction using the Relief algorithm. The top 100 features were selected based on the feature weights. The AUC was regarded as the evaluation index. Six features were selected in the development of the Radiomic ImmunoScore (RIS) model when the AUC reached its peak (more details of this process are described in Additional file 1). The RIS of each cohort was also divided into High-RIS and Low-RIS groups according to the median.

Subsequently, the clinical significance of RIS was displayed intuitively by ROC, and the hazard ratios of each clinicopathological factor were analyzed between the High-RIS and Low-RIS groups.

### Development and validation of the nomogram

We developed a comprehensive model, including RIS and other clinical features, and visualized it with a nomogram. To verify the accuracy and effectiveness of the nomogram, we randomly divided 400 patients from Nanfang Hospital into a training cohort and a validation cohort, with 200 cases in each group.

According to the influence degree of each factor on the outcome variable in the multifactor regression model, each value level of each variable was given a score, and the comprehensive nomogram obtained by adding was calculated by function to attain the prediction probability. Patients were then divided into High-nomogram and low-nomogram groups according to the median nomogram score.

Subsequently, the predictive ability of the nomogram for patients was demonstrated by a variety of evaluation methods, including ROC, survival curve, calibration plot decision curve analysis (DCA), net reclassification improvement (NRI) and risk factor association diagrams.

### Performance of the nomogram in the identification of chemotherapy benefit

The association between the nomogram and adjuvant chemotherapy response was assessed in patients with stage II and III gastric cancer (n = 280, Nanfang cohort). All patients were divided into a chemotherapy cohort (n = 182) and a no chemotherapy cohort (n = 98). Both the total survival time and disease-free survival time were observed. Survival curves were generated with Kaplan–Meier analysis. Hazard ratios were generated with the Cox regression model.

### Statistical analysis

All statistical tests were two-sided, and P < 0.050 was considered statistically significant. Statistical analysis was conducted with R software (version 4.0.4) and SPSS software (version 25.0). The packages for downloading data from TCGA and GEO are TCGAbiolinks and GEOquery. Correlations between the immune cell immune checkpoints and the Immunoscore were analyzed using Pearson’s correlation test. Survival curves were constructed by the Kaplan–Meier method and compared using the log rank test. The sensitivity and specificity of the survival prediction based on the 3 models were depicted by a time-dependent receiver operating characteristic (ROC) curve and a time-independent ROC curve, with quantification of the area under the ROC curve using the timeROC and pROC packages. The results of subgroup analysis were represented by a forest graph using the forestplot package, and the points of HR value in the graph were processed by log2 normalization. We performed Cox multivariate modeling using the rms package. After constructing the nomogram with the nomogramFormula package, we drew decision curves with the rmda package and risk factor correlation diagrams with the ggrisk package to measure the clinical value of the nomogram.

## Results

### Clinical characteristics

Up to 936 GC patients from 4 cohorts (TCGA, GSE62254, GSE15459 and Nanfang Hospital) were enrolled in this study. We collected and analyzed their clinical characteristics, which were summarized in Table 1: 44 patients in the TCGA cohort [38 (86.4%) were male, 6 (13.6%) were female and the mean age was 64.73 ± 9.33 years], 300 patients in the GSE62254 cohort [199 (66.3%) were male, 101 (33.7%) were female and the mean age was 61.94 ± 11.36 years], 192 patients in the GSE15459 cohort [123 (65.1%) were male, 69 (34.9%) were female and the mean age was 64.37 ± 13.24 years], 400 patients in the Nanfang Hospital cohort [276 (69.0%) were male, 124 (31.0%) were female and the mean age was 56.04 ± 10.87 years]. More information on tumors was analyzed and is listed in Table 1, including the depth of invasion, lymph node metastasis, metastasis, TNM stage, pathological type, venous invasion, lymphovascular invasion and perineural invasion.

**Table 1:** Clinicopathologic characteristics of patients

Variables	TCGA Cohort (n = 44)	Nanfang Hospital Cohort (n = 400)	GSE62254 (n = 300)	GSE15459 (n = 192)
Age (years)	64.73 ± 9.33	56.04 ± 10.87	61.94 ± 11.36	64.37 ± 13.24
Sex (male)	38 (86.4%)	276 (69.0%)	199 (66.3%)	125 (65.1%)
T stage				
T1	0	64 (16%)	0	0
T2	1 (2.3%)	39 (9.8%)	188 (62.7%)	0
T3	24 (54.5%)	43 (10.8%)	91 (30.3%)	0
T4	19 (53.2%)	254 (63.5%)	21 (7%)	0
Unknown	0	0	0	192 (100%)
N stage				
N0	8 (18.2%)	144 (36%)	38 (12.7%)	0
N1	9 (20.5%)	76 (19%)	131 (41.7%)	0
N2	12 (27.3%)	72 (18%)	80 (26.7%)	0
N3	14 (31.8%)	108 (27%)	51 (17%)	0
Unknowm	1 (2.3%)	0	0	192 (100%)
M stage				
M0	41 (93.2%)	391 (97.8%)	273 (91%)	0
M1	2 (4.5%)	9 (2.2%)	27 (7%)	0
Unknown	1 (2.3%)	0	0	192 (100%)
Stage				
I	1 (2.3%)	81 (20.3%)	30 (10%)	31 (16.1%)
II	7 (15.9%)	92 (23%)	96 (32.3%)	29 (15.1%)
III	31 (70.5%)	188 (47%)	96 (32%)	72 (37.5%)
IV	4 (9.1%)	25 (12.5%)	77 (25.7%)	60 (31.3%)
Unknown	1 (2.3%)	0	0	0
Pathological_type				
Adenocarcinoma	40 (90.9%)	305 (76.3%)	245 (81.7%)	0
Signet ring cell carcinoma	4 (9.1%)	61 (15.3%)	42 (14%)	0
Others	0	34 (8.5%)	13 (4.3%)	0
Unknown		0	0	192 (100%)
Venous invasion				
Yes	0	326 (81.5%)	44 (14.7%)	0
No	0	74 (18.5%)	129 (43%)	0
Unknown	44 (100%)	0	127 (42.3%)	192 (100%)
Lymphovascular invasion				
Yes	0	340 (85%)	205 (68.3%)	0
No	0	60 (15%)	73 (24.3%)	0
Unknown	44 (100%)	0	22 (17.3%)	192 (100%)
Perineural invasion				
Yes	0	282 (70.5%)	88 (29.3%)	0
No	0	118 (29.5%)	159 (53%)	0
Unknown	44 (100%)	0	53 (17.7%)	192 (100%)
Postoperative chemotherapy				
Yes	0	223 (59.7%)	226 (75.3%)	0
No	0	177 (40.3%)	73 (24.3%)	0
Unknown	44 (100%)	0	1 (0.3%)	192 (100%)

Data are expressed as mean ± standard deviation or number (%)

^a^ According to the 8th edition of the American Joint Committee on Cancer classification

### Derivation and validation of the ImmunoScore (IS)

**Fig. 2:**
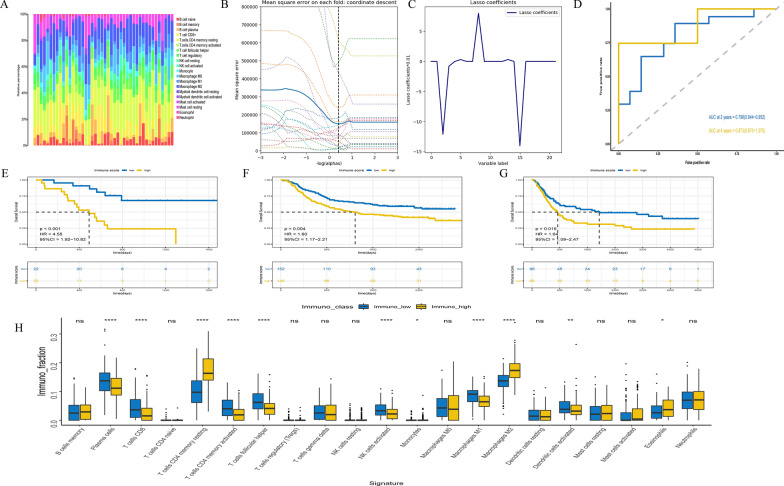
Construction and evaluation of the immunoscore (IS) model. **A** Summary of inferred immune cell subset proportions in the TCGA cohort; **B** Least absolute shrinkage and selection operator (LASSO) coefficient profiles of the 22 immunocytes fractions; **C** Twentyfold cross-validation for tuning parameter selection in the LASSO model; **D** Time-dependent receiver-operating characteristics (ROC) curves for overall survival prediction by the IS model in the TCGA cohort; **E**–**G** Kaplan–Meier curves for overall survival prediction by the IS model in the TCGA cohort, GSE62254 cohort and GSE15459 cohort, respectively; **H** The fraction of tumor microenvironment immune cells in high- and low-IS groups in the GSE62254 cohort

The immunocyte composition of the TME was calculated with RNA sequencing data from the TCGA cohort (Fig. 2A),the GSE62254 cohort (Additional file 3: Figure S1) and the GSE15459 cohort (Additional file 4: Figure S2). As a training cohort, 6 types of immunocytes (plasma cells, CD8 + T cells, memory resting CD4 T cells, Tregs, M2 macrophages, and M0 macrophages) were selected by LASSO Cox regression analysis based on the TCGA cohort (Fig. 2B, C) and calculated the immunoScore by a Cox proportional hazards model (Additional file 2). The prognostic accuracy of the IS was assessed through time-dependent ROC analysis (2-year AUC = 0.798, and 4-year AUC = 0.873, Fig. 2D). The same calculations were performed for GSE62254 and GSE15459 as the validation cohorts (the results are shown in Additional file 2, Additional file 10: Figure S3 and Additional file 11: Figure S4). The survival curves of High-IS and Low-IS groups were generated with Kaplan–Meier analysis [TCGA cohort: p < 0.001, HR = 4.55 (95% CI 1.92–10.82), GSE62254 cohort: p = 0.004, HR = 1.60 (95% CI 1.17–2.21), GSE15459 cohort: p = 0.015, HR = 1.64 (95% CI 1.09–2.47), Fig. 2E–G].

### Relationship between the ImmunoScore (IS) and immune cells and immune checkpoint

Based on the gene expression data of the GSE62254 cohort, we obtained differentially expressed cells from the immune microenvironment (Fig. 2H). Notably, the infiltration of plasma cells, CD8 + T cells, activated CD4 + memory T cells, follicular helper T cells, activated NK cells and M1 macrophages significantly decreased with the higher immune risk score, while the infiltration of resting CD4 + memory T cells and M2 macrophages was significantly higher with the higher IS, which was likely related to the immunosuppressive reaction of the TME. Furthermore, we analyzed the relationship between IS and immune checkpoints, such as PD-1, PD-L1, and CTLA4 (more details are shown in Additional file 5: Table S1). In summary, IS has a strong relationship with immune cells and immune checkpoints.

### Performance of the radiomic ImmunoScore (RIS) Model

**Fig. 3:**
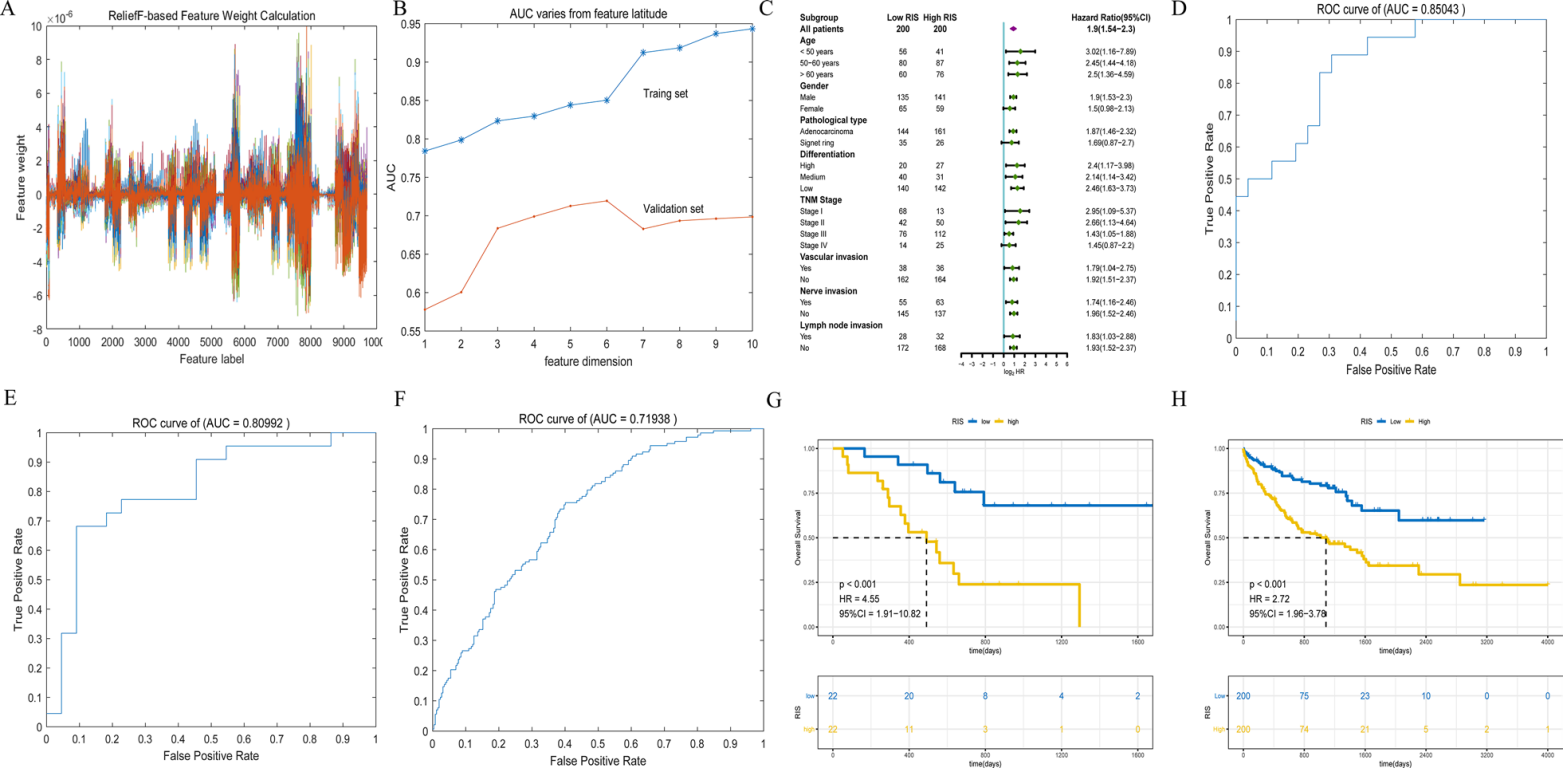
Construction and evaluation of the RIS model. **A** Feature weight based on the relief calculation; **B** Grid search comparing AUCs among various feature dimensions; **C** Hazard Ratios for RIS in each clinicopathological subgroup in the Nanfang hospital cohort; **D**, **E** The performance of RIS model to predict IS measured by ROC curves in the training and validation cohort; **F** The performance of RIS model to predict survival measured by ROC curves in the validation cohort; **G**–**H** Kaplan–Meier curves for patients with High- and Low-RIS in the training and validation cohort

As a training cohort from TCGA, radiomics signatures were compared with 44 cases based on the CT image data, which showed the best effect when 6 features were included (Fig. 3A, B). The ability of the RIS model to distinguish High-IS or Low-IS accurately and predict prognosis exhibited an AUC of 0.85 (Fig. 3D) and 0.81 (Fig. 3E), respectively. The ability of RIS to predict survival had an AUC of 0.72 (Fig. 3F) in the validation cohort from Nanfang Hospital. In the training cohort, overall survival curves were generated with Kaplan–Meier analysis, comparing High-RIS with Low-RIS [p < 0.001, HR = 4.55 (95% CI 1.91–10.82), Fig. 3G], and the result was similar in the validation cohort [p < 0.001, HR = 2.72 (95% CI 1.96–3.78), Fig. 3H]. Furthermore, in the forest plot of the Nanfang Hospital cohort, the significant Hazard Ratios were found for RIS in each subgroup, more specifically, in each age stage (< 50, 50–65, > 65), each differentiation stage (low, medium, high), and regardless of vascular invasion, nerve invasion and lymph node invasion (Fig. 3C).

### Development, comparison and validation of the nomogram

**Fig. 4:**
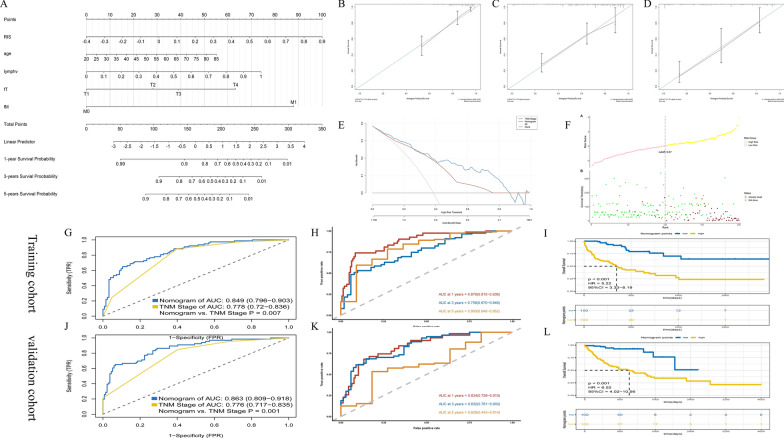
Evaluation of nomogram integrated RIS and clinical pathological variables in the training cohort. **A** Nomogram for predicting the ratio of GC patients with a certain survival time in the training cohort; Calibration plots describing the calibration of nomogram based on the consistency between predicted and observed 1-year (**B**), 3-year (**C**) and 5-year (**D**) results; **E** Decision curves comparing the nomogram and TNM stage among a series of risk thresholds; **F** Risk factor association diagram showing the distribution of prognosis of each patient with high- or low-nomogram scores; **G**, **J** Time-independent ROC curves comparing the predictive accuracy of nomogram and TNM stage; **H**, **K** Time-dependent ROC curves comparing the predictive accuracy of nomogram and TNM stage; **I**, **L** Kaplan–Meier analysis of overall survival curves of High- and Low-nomogram in training group and validation group

A nomogram was developed by Cox regression based on the RIS and clinical features (Fig. 4A), and the Cox regression coefficient and corresponding score are shown in Additional file 6: Table S2. From the results of univariate survival analysis, RIS, tumor metastasis, lymph node positive detection rate, T stage and age were significant factors associated with OS and DFS (Additional file 7: Table S3, Additional file 8: Table S4). By calculating the total score and finding the corresponding prediction probability on the total point scale, the 1-year, 3-year and 5-year estimated survival probability could be obtained. Based on calibration plots for predicting 1-year, 3-year and 5-year survival (Fig. 4B–D), the predicted calibration curve was near the standard curve, or the 45-degree line, demonstrating that the derived nomogram had considerable prediction capability. Likewise, the decision curve analysis (DCA) depicted in Fig. 4E indicated that the nomogram had a higher net income than traditional TNM among a series of risk thresholds. The results of comparing the nomogram with the TNM staging system by Net Reclassification Improvement (NRI) are as follows: 1-year (NRI = 0.27, 95% CI = 0.07–0.44), 3-year (NRI = 0.03, 95% CI =  − 0.17–0.18), and 5-year (NRI = 0.05, 95% CI =  − 0.26–0.26), indicating that the nomogram had a relatively accurate prognosis. Simultaneously, the risk factor association diagram (Fig. 4F) showed the distribution difference of prognosis between High-nomogram and Low-nomogram groups distinguished by the nomogram. To predict the survival of GC patients, the performance of the nomogram was better than that of TNM staging (training cohort: nomogram AUC = 0.849 and TNM staging system AUC = 0.778, p = 0.007, Fig. 4G; validation cohort: nomogram AUC = 0.863 and TNM staging system AUC = 0.776, p = 0.001, Fig. 4J). The results of the time-dependent ROC curve analysis in the training cohort and validation cohort are shown in Fig. 4H, K. Comparing the High-nomogram and Low-nomogram groups, the survival curves of the training cohort and validation cohort are shown in Fig. 4I, L, which indicated that the prognosis of the Low-nomogram group was better than that of the High-nomogram group.

### Identification of chemotherapy beneficiaries with a nomogram

**Fig. 5:**
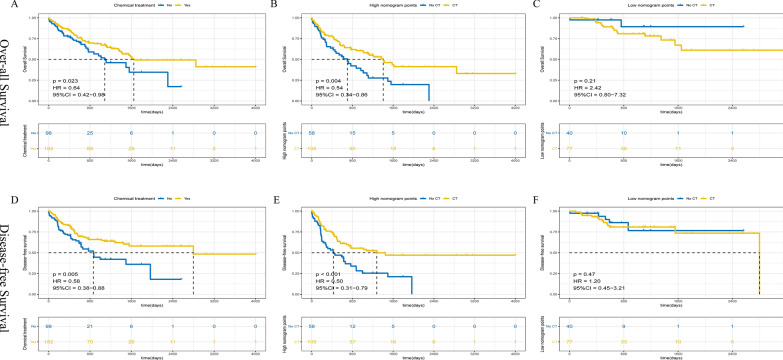
Survival impact of nomogram score among TNM stage II and III subsets. Kaplan–Meier curves for overall survival (**A**–**C**) and disease-free survival (**D**–**F**) of the chemotherapy group and non-chemotherapy group in the whole cohort (**A**, **D**), high nomogram score subset (**B**, **E**) and low nomogram score subset (**C**, **F**)

In addition, we analyzed differences in chemotherapy benefit among TNM stage II and III GC patients with different groups based on the nomogram. Chemotherapy dose leads to survival benefit for GC patients (OS: p = 0.023, and DFS: p = 0.005), but the degree of benefit varied among patients in different nomogram groups (High-nomogram group and Low-nomogram group) (Fig. 5). In the High-nomogram group, GC patients could significantly benefit with chemotherapy regarding OS and DFS (OS: p = 0.004, and DFS: p < 0.001), while in the Low-nomogram group, chemotherapy did not provide a survival benefit to patients compared with no chemotherapy (OS: p = 0.21, and DFS: p = 0.47). Furthermore, we analyzed the interactions between chemotherapy and death or recurrence in the High-nomogram and Low-nomogram groups with Cox proportional hazards regression, and High-nomogram patients benefited more from chemotherapy [OS: HR 0.41 (0.26–0.64), p < 0.001; DFS: HR 0.54 (0.34–0.84), p < 0.001] than Low-nomogram patients (Additional file 9: Table S5). In summary, High-nomogram GC patients benefit more from chemotherapy than Low-nomogram GC patients.

## Discussion

Accurate assessment of the prognosis and beneficial choices for treating GC patients are essential for clinicians. Identifying an evidence-based and broadly utilized classification system for GC patients is challenging. Since its development, the AJCC TNM staging system has been widely used in the prognosis assessment of tumors and in guiding the selection of treatment regimens [21]. However, as we learn more about the tumor microenvironment, we realize this staging system has inevitable limitations, including that it is difficult to explain the complex biological behavior of the tumor based on tumor invasion depth, lymph node metastasis, and distant organ metastasis only. In the same TNM stage, patients may have different outcomes, while patients in different TNM stages may have similar outcomes [22–24]. An increasing number of studies have found that the infiltration of immunocytes in the TME is closely related to the prognosis and treatment sensitivity of patients [25, 26]. Furthermore, immune checkpoint inhibitors have shown positive results in therapies for cancer, such as ipilimumab targeting CTLA4 [27] and lambrolizumab targeting PD-1 [28].

The individualized analysis of the TME is a major advance in the history of fighting against cancer, and it has also led to the development of many methods for analyzing the immune microenvironment, such as single-cell sequencing (SNS) [29, 30] and immunohistochemistry (IHC) [1, 3]. The CIBERSORT algorithm is a method applied to calculate the proportion of immunocytes in the tumor microenvironment based on next-generation sequencing (NGS) data [17, 31]. In our study, we developed a method to calculate the immune score to assess the immunocyte infiltration of the TME based on NGS data from TCGA, and the method was validated in 2 cohorts from GEO.

We developed a further model (RIS) by SVM to assess the immune score of the TME, which established a bridge between CT images and NGS. The RIS was also validated in GC patients from the Nanfang Hospital cohort. The AUC value was significantly higher than that of the TNM staging system in assessing the prognosis of GC patients.

Using SNS, IHC and NGS may be effective in assessing immunocyte infiltration of the TME. However, these methods are costly for patients and complex for clinicians, especially in remote areas with underdeveloped medical resources, and the contradictions will become more obvious. Moreover, IHC staining of tissue slices to evaluate the immune environment might not be accurate due to the subjectivity of observers and the limitation of hysteresis.

Medical images are data [8], such as radiomics, which has been developed and widely used in learning more information about tumors [32, 33]. Because the characteristics of radiomics are complex and diverse, we used machine learning to identify the radiomics characteristics of patients with different immune scores rapidly and effectively. The SVM is a mature machine learning method that has demonstrated excellent performance in multifactor classification and survival prediction. Based on the RIS model, we can quickly obtain a patient's individual immune score from his/her CT images, which means that this RIS model can greatly improve the efficiency of clinicians in evaluating patients' TME and greatly reduce the high cost of sequencing for patients.

Prognosis is related not only to the TME of the tumor but also to the clinical characteristics of the patients. To improve the ability of the RIS model to evaluate the prognosis of patients and maximize the prognostic value of clinical characteristics, we combined the RIS and clinical characteristics by Cox regression and visualized it in a nomogram. Based on the nomogram, we can more easily and intuitively calculate the individual 1-year, 3-year and 5-year survival rates of patients.

Adjuvant chemotherapy is now considered a common treatment for stage II and III GC patients, improving their survival in two large-scale trials [34, 35] (the ACTS-GC trial and CLASSIC trial). However, some researchers noted that the OS benefit was moderate with adjuvant chemotherapy, meaning that not all GC patients could benefit from adjuvant chemotherapy [36]. The identification of adjuvant chemotherapy benefits is still a challenging task for researchers [37]. In our study, based on the nomogram, High-nomogram GC patients could benefit significantly more from adjuvant chemotherapy.

Due to the universality of CT scanning and the availability of clinical features, we can quickly and effectively obtain TME assessment, prognosis and adjuvant chemotherapy benefit of GC patients based on our RIS model and nomogram. The strategy that we have developed for evaluating the TME of GC patients is useful and easy to implement, especially for clinicians of grassroots community hospitals, in the management and treatment of GC patients.

At the same time, we have also noticed that many researchers developed novel theranostics trying to use in cancer treatment, such as nanomaterials [38, 39]. For the potential of nanomaterials to deliver, enhance, and finetune in treatment, it shows exciting effects in animal cancer models, which may be a kind of hopeful treatments in the future [40]. However, it’s difficult to identify who would benefit in novel theranostics and easily assess the effectiveness of novel theranostics. Maybe, AI could be the solution to these bottleneck problems. For example, Tang et.al developed a AI model to analyse the distribution of intratumoral nanoparticles to optimize the cancer treatment [41]. It's worth noting that antioxidation therapy is an important mechanism of nanomaterials in treating with cancer [42]. It works by reducing tumor-promoting oxidative stress and remodeling the TME. Perhaps, our AI model could reflect the antitumor efficacy of nanomaterials by analyzing TME in real-time.

There were several limitations to this study. First, the number of GC patients enrolled in the TCGA cohort was small due to the limited number of cases with CT images. Second, due to the limitations of data acquisition, the data types contained in the different cohorts were not perfect, but this did not affect our RIS model and nomogram development. We hope that more data can be obtained to develop and validate our RIS model and nomogram. Third, the study was limited to collecting retrospective data, and we need further prospective research to validate our strategy in assessing TME, prognosis and adjuvant chemotherapy benefit.

In conclusion, the RIS model and the nomogram can be used to assess the TME, prognosis and adjuvant chemotherapy benefit of GC patients after radical gastrectomy and seems to be a valuable addition to the current TNM staging system. High-nomogram GC patients may benefit more from adjuvant chemotherapy than low-nomogram GC patients. By enrolling more GC patients for validation, we hope our strategy will provide more individualized assistance to clinicians and patients.

## Supplementary Information

**Additional file 1**: The process of features selecting at highest AUC.

**Additional file 2**: The immunoScore was calculated of TCGA,GSE62254 and GSE15459 cohorts.

**Additional file 3**: The immunocyte composition of the TME of GSE62254 cohort.

**Additional file 4**: The immunocyte composition of the TME of GSE15459 cohort.

**Additional file 5**: Correlation analyses between Immunoscore and gene expressions and immunocytes.

**Additional file 6**: Cox regression coefficients and nomogram score for the training cohort.

**Additional file 7**: Univariable analysis of the RIS, clinical features with overall survival in Nanfang cohort.

**Additional file 8**: Univariable analysis of the RIS, clinical features with disease-free survival in Nanfang cohort.

**Additional file 9**: Treatment Interaction with Nomogram for DFS and OS with Stage II and III Disease.

**Additional file 10**: Time-dependent ROC analysis of GSE62254 cohort.

**Additional file 11**: Time-dependent ROC analysis of GSE15459 cohort.

## Data Availability

All data generated or analyzed during this study are included either in this paper or in the additional information. All readers can obtain the original code of the SVM model and the raw data of clinical trials in this study by contacting us.
