# Supplementary material for: An artificial intelligence method to assess the tumor microenvironment with treatment outcomes for gastric cancer patients after gastrectomy
Source: J Transl Med. 2022 Feb 21;20:100. doi: 10.1186/s12967-022-03298-7 (PMC8862309; doi:10.1186/s12967-022-03298-7)
Supplement: Supplementary file 1 — Additional file 1: The process of features selecting at highest AUC. [file 12967_2022_3298_MOESM1_ESM.docx]

**The relief of CT features**

For dimension reduction, we use the Relief algorithm to randomly take a sample R from training cohort each time, subsequently find K near hit samples of R from the same kind one and K near miss samples from different one. The weight of each feature was then updated according to the following rules: if the distance of R and near hit on a feature was smaller than that on R and near miss, it illustrated that the feature was beneficial to distinguish the nearest neighbors of the same kind and different classes, so we increased the weight of the feature; otherwise we reduced that. The above process was repeated for m times, and finally the average weight of each feature was obtained.

Select the top100 features of feature weight ranking, and take the first one as the first feature of SVR regression model. We then calculated the AUC of the training cohort after cross testing for this feature; gradually increased the order of the model forward until all feature combinations were traversed to find which combination AUC was the largest when finding the first feature bit, afterwards repeat this process to fine the optimal subset.
