# Supplementary figures and images for: An artificial intelligence method to assess the tumor microenvironment with treatment outcomes for gastric cancer patients after gastrectomy
Source: J Transl Med. 2022 Feb 21;20:100. doi: 10.1186/s12967-022-03298-7 (PMC8862309; doi:10.1186/s12967-022-03298-7)

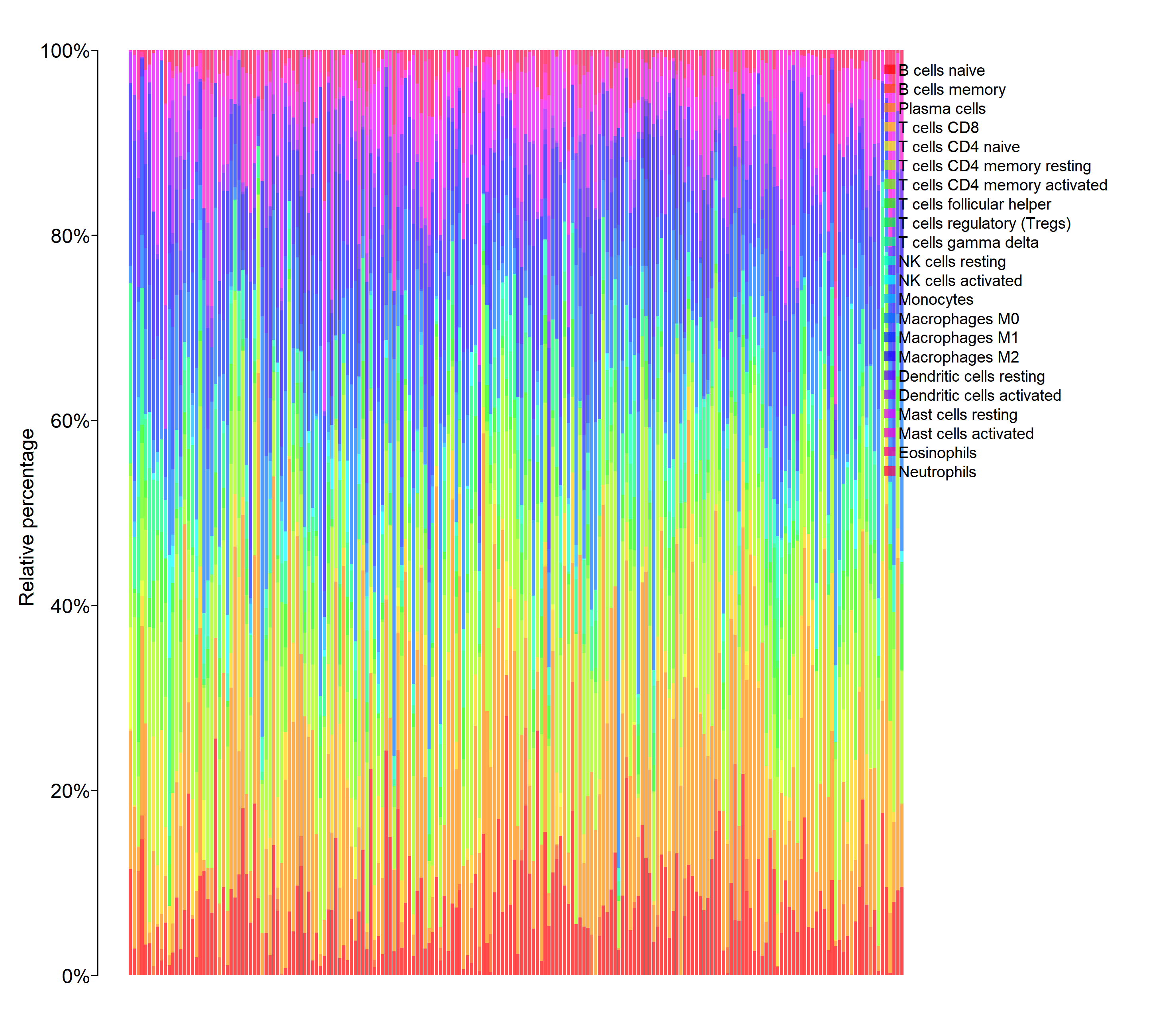

Supplement: Supplementary file 3 — Additional file 3: The immunocyte composition of the TME of GSE62254 cohort. [file 12967_2022_3298_MOESM3_ESM.tiff]

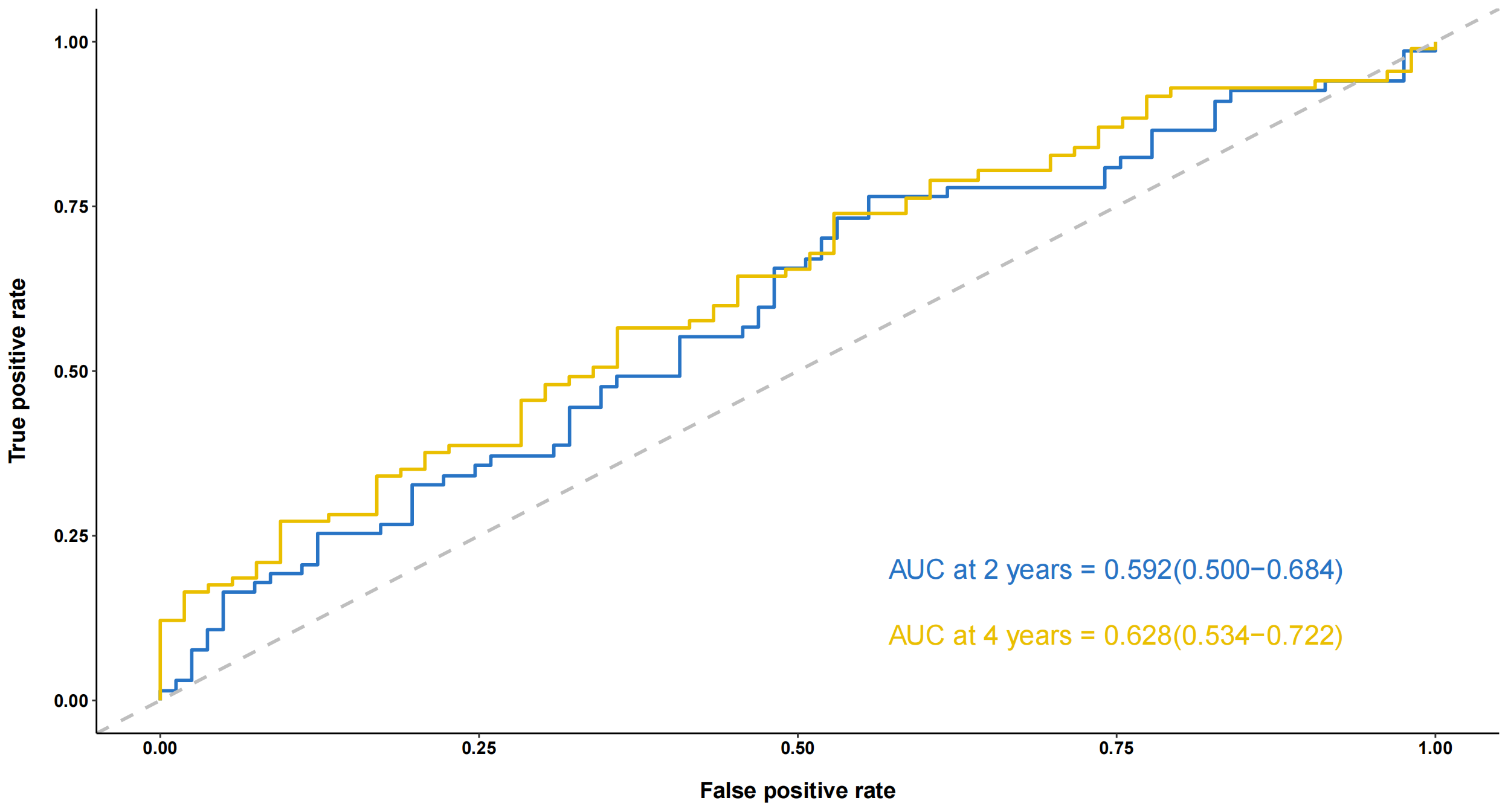

Supplement: Supplementary file 10 — Additional file 10: Time-dependent ROC analysis of GSE62254 cohort. [file 12967_2022_3298_MOESM10_ESM.tif]

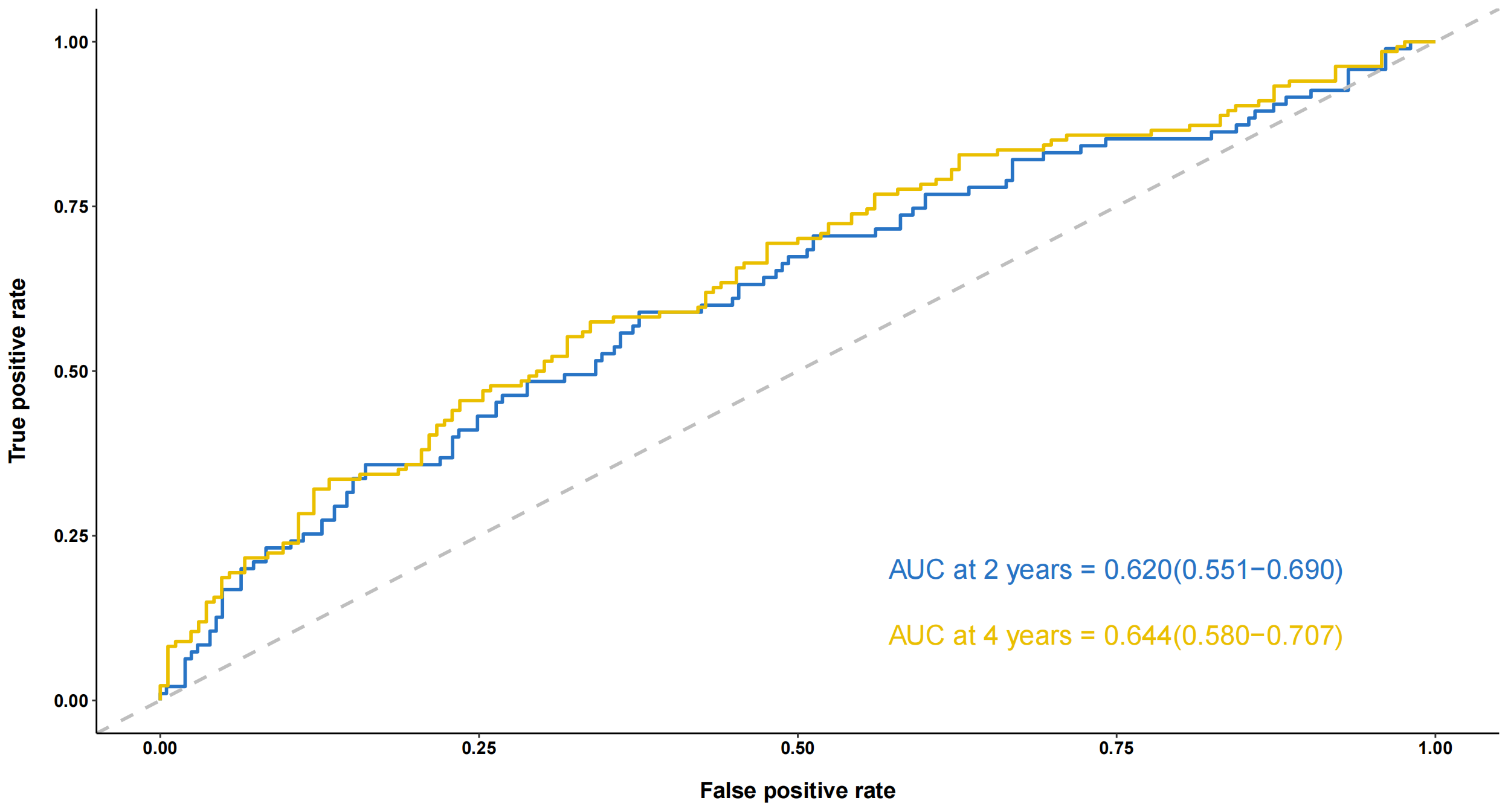

Supplement: Supplementary file 11 — Additional file 11: Time-dependent ROC analysis of GSE15459 cohort. [file 12967_2022_3298_MOESM11_ESM.tif]
