# Supplementary material for: An artificial intelligence method to assess the tumor microenvironment with treatment outcomes for gastric cancer patients after gastrectomy
Source: J Transl Med. 2022 Feb 21;20:100. doi: 10.1186/s12967-022-03298-7 (PMC8862309; doi:10.1186/s12967-022-03298-7)
Supplement: Supplementary file 5 — Additional file 5: Correlation analyses between Immunoscore and gene expressions and immunocytes. [file 12967_2022_3298_MOESM5_ESM.docx]

**TABLE . Correlation analyses between Immunoscore and gene expressions and immunocytes**

| **Features** | **Genes/Immunocytes** | **r** | **95%CI** | **P-value** |
| --- | --- | --- | --- | --- |
| Immune checkpoint regulators | CD274 | -0.31 | -0.41~-0.20 | 0.000 |
|  | CTLA4 | -0.14 | -0.25~-0.02 | 0.019 |
|  | ERBB2 | -0.16 | -0.27~-0.05 | 0.004 |
|  | PDCD1 | -0.23 | -0.34~-0.12 | 0.000 |
| Immunocytes | CD8 T cells | -0.18 | -0.30~-0.04 | 0.009 |
|  | Activated NK cells | -0.25 | -0.36~-0.13 | 0.000 |
|  | Activated CD4 memory T cells | -0.37 | -0.48~-0.25 | 0.000 |
|  | Plasma cells | -0.47 | -0.55~-0.37 | 0.000 |
|  | T follicular helper cells | -0.38 | -0.48~-0.28 | 0.000 |
|  | M1 macrophage | -0.17 | -0.28~-0.06 | 0.004 |
|  | Resting CD4 memory T cells | 0.58 | 0.50~0.65 | 0.000 |
|  | M2 macrophage | 0.67 | 0.60~0.72 | 0.000 |

Abbreviations:CI, confidence interval
