# Supplementary material for: An artificial intelligence method to assess the tumor microenvironment with treatment outcomes for gastric cancer patients after gastrectomy
Source: J Transl Med. 2022 Feb 21;20:100. doi: 10.1186/s12967-022-03298-7 (PMC8862309; doi:10.1186/s12967-022-03298-7)
Supplement: Supplementary file 6 — Additional file 6: Cox regression coefficients and nomogram score for the training cohort. [file 12967_2022_3298_MOESM6_ESM.docx]

**Table S2 Cox regression coeﬃcients and nomogram score for the training cohort**

|  | **Cox regression coeﬃcients** | **Nomogram score** |
| --- | --- | --- |
| **RIS^a^** | **1.70** | **76.92*RIS + 30.77** |
| **Age^a^** | **0.02** | **0.8712*Age-17.4236** |
| **Lymphv^a^** | **1.81** | **8.251e+01*Lymphv+ 8.569e-15** |
| **fT** |  |  |
| **T1** | **0** | **0** |
| **T2** | **0.72** | **24** |
| **T3** | **1.02** | **48** |
| **T4** | **1.64** | **72** |
| **fM** |  |  |
| **M0** | **0** | **0** |
| **M1** | **2.17** | **99** |

**^a^ Continuous variable**
