# Supplementary material for: An artificial intelligence method to assess the tumor microenvironment with treatment outcomes for gastric cancer patients after gastrectomy
Source: J Transl Med. 2022 Feb 21;20:100. doi: 10.1186/s12967-022-03298-7 (PMC8862309; doi:10.1186/s12967-022-03298-7)
Supplement: Supplementary file 7 — Additional file 7: Univariable analysis of the RIS, clinical features with overall survival in Nanfang cohort. [file 12967_2022_3298_MOESM7_ESM.docx]

| Table S3. Univariable analysis of the RIS, clinical features with overall survival in Nanfang cohort. | | | |
| --- | --- | --- | --- |
| **Variable** | **Nanfang cohort**  **(N=400)** | | |
|  | Hazard.Ratio | CI 95% | *P* |
| Age^a^(years) | 1.026 | 1.010-1.043 | 0.001 |
| Diameter^a^ | 1.003 | 0.999-1.006 | 0.057 |
| Depth of invasion |  |  |  |
| T2 vs. T1 | 2.764 | 0.8080-9.455 | 0.105 |
| T3 vs. T1 | 5.825 | 1.876-18.086 | 0.002 |
| T4 vs. T1 | 8.919 | 3.292-24.166 | <0.001 |
| Lymph node metastasis |  |  |  |
| N1 vs. N0 | 1.818 | 0.970-3.409 | 0.062 |
| N2 vs. N0 | 3.837 | 2.185-6.738 | <0.001 |
| N3 vs. N0 | 7.012 | 4.217-11.659 | <0.001 |
| Distant metastasis |  |  |  |
| M1 vs. M0 | 8.240 | 3.977-17.070 | <0.001 |
| Clinical stage |  |  |  |
| Ⅱ vs. Ⅰ | 2.248 | 0.809-6.242 | 0.120 |
| Ⅲ vs. Ⅰ | 8.580 | 3.486-21.114 | <0.001 |
| Ⅳ vs. Ⅰ | 43.825 | 16.913-113.558 | <0.001 |
| Lymphovascular invasion | 13.780 | 8.498-22.350 | <0.001 |
| **RIS^a^** | **13.220** | **5.627-31.040** | **<0.001** |
|  |  |  |  |

^a^Continuous variable

Abbreviations:HR, hazard ratio;CI, confidence interval.
