# Supplementary material for: An artificial intelligence method to assess the tumor microenvironment with treatment outcomes for gastric cancer patients after gastrectomy
Source: J Transl Med. 2022 Feb 21;20:100. doi: 10.1186/s12967-022-03298-7 (PMC8862309; doi:10.1186/s12967-022-03298-7)
Supplement: Supplementary file 8 — Additional file 8: Univariable analysis of the RIS, clinical features with disease-free survival in Nanfang cohort. [file 12967_2022_3298_MOESM8_ESM.docx]

| Table S4. Univariable analysis of the RIS, clinical features with disease-free survival in Nanfang cohort. | | | |
| --- | --- | --- | --- |
| **Variable** | **Nanfang cohort**  **(N=400)** | | |
|  | Hazard.Ratio | CI 95% | *P* |
| Age^a^(years) | 1.015 | 0.999-1.031 | 0.060 |
| Diameter^a^ | 0.999 | 0.996-1.004 | 0.971 |
| Depth of invasion |  |  |  |
| T2 vs. T1 | 1.070 | 0.380-3.012 | 0.897 |
| T3 vs. T1 | 3.357 | 1.515-7.437 | 0.003 |
| T4 vs. T1 | 3.952 | 2.002-7.800 | <0.001 |
| Lymph node metastasis |  |  |  |
| N1 vs. N0 | 1.164 | 0.708-1.914 | 0.548 |
| N2 vs. N0 | 1.099 | 1.322-3.334 | 0.002 |
| N3 vs. N0 | 1.911 | 1.248-2.925 | 0.003 |
| Distant metastasis |  |  |  |
| M1 vs. M0 | 9.653 | 4.554-20.460 | <0.001 |
| Clinical stage |  |  |  |
| Ⅱ vs. Ⅰ | 6.668 | 2.846-15.620 | <0.001 |
| Ⅲ vs. Ⅰ | 4.920 | 2.117-11.440 | <0.001 |
| Ⅳ vs. Ⅰ | 56.652 | 23.269-137.930 | <0.001 |
| Lymphovascular invasion | 3.251 | 1.857-5.691 | <0.001 |
| **RIS^a^** | **14.870** | **6.354-34.780** | **<0.001** |
|  |  |  |  |

^a^Continuous variable

Abbreviations:HR, hazard ratio;CI, confidence interval.
