# Supplementary material for: An artificial intelligence method to assess the tumor microenvironment with treatment outcomes for gastric cancer patients after gastrectomy
Source: J Transl Med. 2022 Feb 21;20:100. doi: 10.1186/s12967-022-03298-7 (PMC8862309; doi:10.1186/s12967-022-03298-7)
Supplement: Supplementary file 9 — Additional file 9: Treatment Interaction with Nomogram for DFS and OS with Stage II and III Disease. [file 12967_2022_3298_MOESM9_ESM.docx]

**SUPPLEMENTARY TABLE 5. Treatment Interaction with Nomogram for DFS and OS with Stage Ⅱ and Ⅲ Disease.**

|  | **DFS** | |  | **OS** | |  |
| --- | --- | --- | --- | --- | --- | --- |
| **Stage II and III（n=280)** | **CT vs No CT HR**  **(95% CI)** | **P** | **P value for interaction** | **CT vs No CT HR**  **(95% CI)** | **P** | **P value for interaction** |
| **Low RIS** | **2.29（0.92-5.67）** | **0.159** | **0.080** | **1.72(0.68-4.33)** | **0.251** | **0.025** |
| **High RIS** | **0.54(0.34-0.84)** | **<0.001** |  | **0.41(0.26-0.64)** | **<0.001** |  |

Abbreviation: HR, hazard ratio; CI, confidence interval
